# “Recurrent multiple cerebral infarctions related to the progression of adenomyosis: a case report”

**DOI:** 10.1186/s12883-018-1117-1

**Published:** 2018-08-21

**Authors:** Yasuhiro Aso, Ryo Chikazawa, Yuki Kimura, Noriyuki Kimura, Etsuro Matsubara

**Affiliations:** Department of Neurology, Faculty of Medicine, Oita University, Yufu-city, Oita 879-5593 Japan

**Keywords:** Multiple cerebral infarctions, Adenomyosis, Hypercoagulagulability, Anticoagulation therapy

## Abstract

**Background:**

Benign gynecologic tumor, such as uterine adenomyosis, has been suggested to develop hypercoagulability. Although some cases of cerebral infarction associated with adenomyosis have been reported, the mechanism of hypercoagulation initiated by adenomyosis is still not clear, and the therapeutic strategy is uncertain.

**Case presentation:**

A 44-year-old woman was presented to our department with headache, left hand weakness, and gait disturbance during her menstrual phase. She had a history of adenomyosis and infertility treatment for 18 years and heavy menstrual bleeding. Magnetic resonance imaging on admission showed multiple hyperintense lesions in cortical and subcortical areas in the cerebrum and cerebellum on diffusion-weighted imaging. Transesophageal echocardiography showed neither embolic sources nor existence of foramen ovale. Her laboratory data revealed anemia, a high D-dimer level, and elevated levels of a mucinous tumor marker. She had adenomyosis and no malignancy was detected. Anticoagulation therapy with intravenous heparin followed by rivaroxaban did not prevent recurrence of cerebral infarction. We discontinued rivaroxaban, and started warfarin therapy with pseudomenopause treatment, which prevented recurrence for 6 months. Five months after her last pseudomenopause treatment, multiple cerebral infarctions occurred. Total hysterectomy was performed, which prevented recurrence of the multiple cerebral infarctions for 2 years without anticoagulation therapy.

**Conclusions:**

Our findings reveal for the first time that anticoagulation therapy, including novel oral anticoagulants, had no preventive effect against cerebral infarctions associated with adenomyosis in a middle-aged woman. Although pseudomenopause treatment temporarily prevented recurrence, resection of the adenomyosis might be the most effective therapy in these cases.

## Background

Uterine adenomyosis is a benign gynecologic condition, defined as the presence of ectopic endometrial glands and stroma surrounded by hyperplastic smooth muscle within the myometrium. Approximately 20% of women attending a general gynecologic clinic were revealed to have adenomyosis [1]. Although common symptoms are menorrhagia, dysmenorrhea, and heavy menstrual bleeding, one-third of women are asymptomatic [2].

Some patients with adenomyosis develop multiple cerebral infarctions (CIs) [3–5] (Table 1). Almost all patients are middle-aged, with severe anemia and elevated serum carbohydrate antigen 125 (CA125). These patients are initially administered conventional anticoagulant therapy, which is often combined with gonadotropin-releasing hormone (GnRH) therapy, and in some cases the adenomyosis is subsequently resected.

**Table 1:** Patient characteristics and therapy for CI associated with adenomyosis

Case	Age (y.o.)	D-dimer (μg/ml)	FDP (μg/ml)	CA125 (U/ml)	hemoglobin (g/dl)	treatment for CI		treatment for adenomyosis	recurrence of CI	reference
						initial treatment	subsequent treatment			
1	45	1.1	–	159	8.4	hepalin	antipletelet therapy	GnRH agonist	–	Yamashiro K et al. 2012
2	44	–	5.9	–	7	hepalin	warfalin	GnRH agonist	–	
3	50	0.57	–	42.6	6.9	aspirin	–	GnRH agonist	–	
4	42	6.0	–	1750	8.6	antiplatelet therapy	warfalin	GnRH agonist	+	
5	59	7.0	–	334.8	–	antithrombotic therapy	–	–	–	Hijikata N et al. 2016

Here, we report the case of a middle-aged woman with adenomyosis who developed multiple CIs in her menstrual phase. Although she received edaravone and anticoagulation treatment with rivaroxaban, the recurrence of multiple CIs was not prevented. Pseudomenopause therapy with a GnRH agonist normalized her hypercoagulation state, but multiple CIs occurred after therapy was discontinued. Finally, a hysterectomy was performed, which successfully prevented CI recurrence. We propose that treatment of CIs due to adenomyosis with anticoagulant therapy is not effective, and briefly discuss the underlying etiology and therapeutic strategy.

## Case presentation

**Fig. 1:**
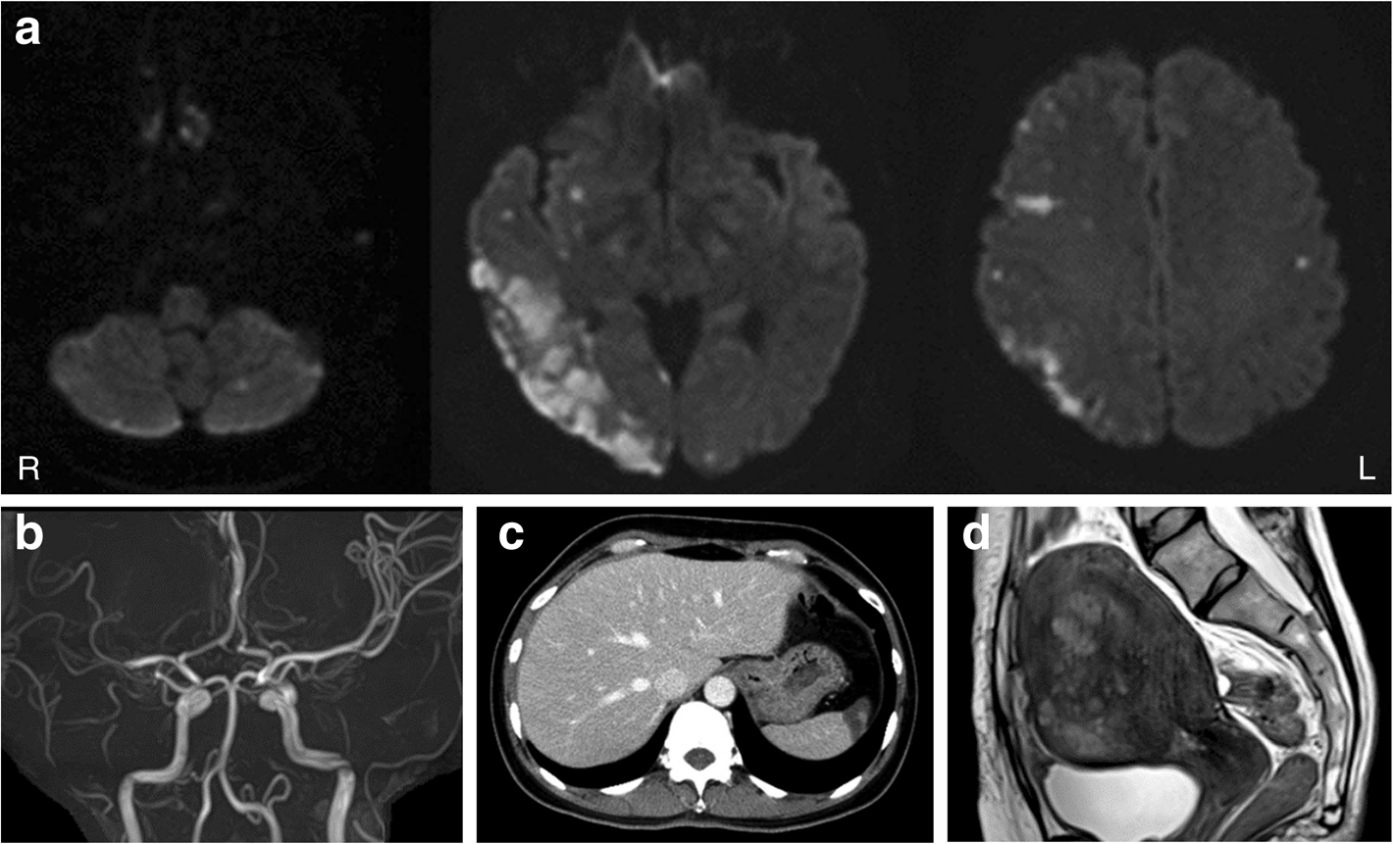
Diffusion-weighted magnetic resonance imaging (MRI) scan of the brain reveals multiple infarctions in the cerebellum and cerebrum (**a**). The M2, M3, and M4 portions of the right middle cerebral artery were not well visualized by MR angiography (**b**). Contrast computed tomography revealed splenic infarction (**c**). Giant adenomyosis was revealed by a T2-weighted MRI scan of the pelvis (**d**)

A 44-year-old woman experienced sudden onset of difficulty using her left hand and walking during her menstrual phase. She had a history of adenomyosis and infertility treatment for 18 years, and heavy menstrual bleeding. She complained of headache, abdominal pain, nausea, and had a fever (37.7 °C) at presentation. She is not obese (BMI of 21.5 kg/m^2^), had no history of taking steroids or contraceptives. Neurologic examination revealed left spatial neglect, left facial hypoalgesia, mild paresis in her left arm, and right pyramidal signs. Brain magnetic resonance imaging (MRI) revealed bilaterally multiple infarctions in the cerebrum and cerebellum, including cortical and subcortical lesions (Fig. 1a). MR angiography presented severe stenosis in the M2, M3, >and M4 portions of right middle cerebral artery (Fig. 1b). Contrast computed tomography revealed a splenic infarction (Fig. 1c). Blood examination revealed normocytic anemia (hemoglobin 10.3 g/dl, mean corpuscular volume 90.5 μm^3^), thrombocytopenia (112,000 /μl), and low-grade elevation of C-reactive protein (2.9 mg/dl). The serum levels of D-dimer (17.0 μg/ml, normal < 0.5 μg/ml), CA125 (2115 U/ml, normal < 35.0 U/ml), and carbohydrate antigen 19–9 (CA19–9) (1824 U/ml, normal < 37.0 U/ml) were increased. Results of a hypercoagulable panel, including protein C and S, antithrombin Ш level, lupus anticoagulant, and anticardiolipin antibody titers, were within normal limits. Pelvic MRI revealed giant adenomyosis (Fig. 1d), but no malignancy was detected. Fluorine-18–2-fluoro-2-deoxy-d-glucose (FDG) positron emission tomography revealed FDG accumulation in the adenomyosis, but no malignancy was detected by cervical cytology. The result of continuous electrocardiography monitoring, transesophageal echocardiography with agitated saline injection, carotid ultrasonography, upper gastrointestinal endoscopy, and colonoscopy were normal.

She was treated with edaravone (60 mg/day) and anticoagulated with heparin for 2 weeks. Subsequently, she was treated with rivaroxaban (15 mg/day). Her serum levels of D-dimer, CA125, and CA19–9 improved (2.7 μg/ml, 911 U/ml, and 501 U/ml, respectively), and the treatment was continued.

**Fig. 2:**
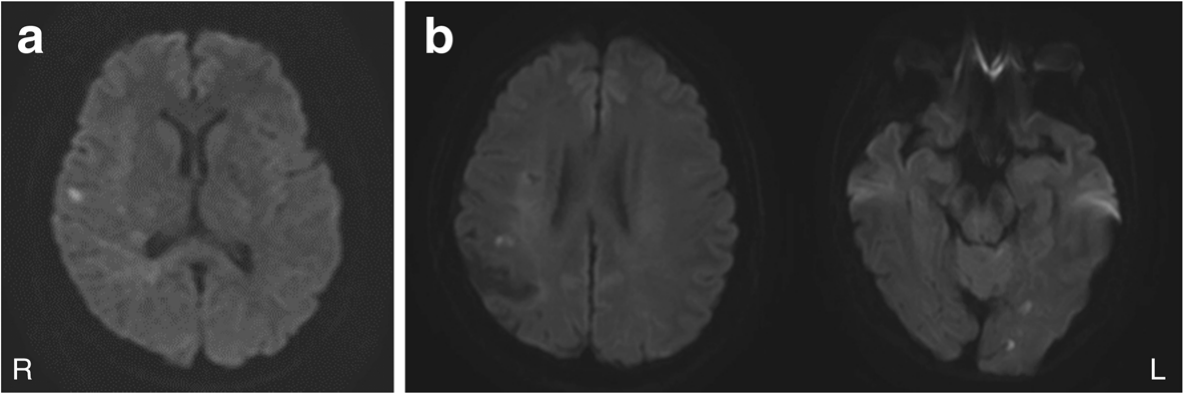
Diffusion-weighted MRI revealed CIs in the right cerebrum (**a**). Multiple cortical and subcortical CIs in the left occipital lobe and right parietal lobe (**b**)

At day 31, a day after her menstrual phase started, she complained of numbness in her left lower limb. Brain MRI revealed a new CI in the right cerebrum (Fig. 2a). The serum levels of D-dimer, CA125, CA19–9 were 2.4 μg/ml, 561 U/ml, 417 U/ml, respectively. We discontinued the rivaroxaban treatment, and started anticoagulant therapy with warfarin. Afterwards, her menstrual bleeding increased, the anemia progressed, and the serum level of D-dimer increased (14.1 μg/ml). She started to receive pseudomenopause treatment with a GnRH agonist for the adenomyosis. Ten days after initiating the GnRH agonist treatment, her serum D-dimer level improved (2.30 μg/ml). She continued the warfarin and GnRH agonist once a month for 6 months, and showed no recurrence of CIs during that time. The serum levels of CA125, CA19–9 improved after 3 months of initiating the therapy (117 U/ml, 224 U/ml, respectively).

She presented with transient weakness of her right lower limb and visited our clinic 5 months after her last GnRH agonist therapy, when her irregular menstrual bleeding had continued for a month. Brain MRI revealed new multiple cortical and subcortical infarctions in the left occipital lobe and right parietal lobe (Fig. 2b). Her serum D-dimer, FDP, CA125, and CA19–9 levels were elevated (22.0 μg/ml, 56.5 μg/ml, 1291.6 U/ml, and 803.2 U/ml, respectively). Anticoagulant therapy with warfarin was well controlled (PT-INR 2.5), and her electrocardiographic findings were normal. We concluded that total hysterectomy would be the most effective therapy for preventing CI recurrence. She underwent total hysterectomy and bilateral salpingo-oophorectomy, which was effective in not only preventing CI recurrence, but also for normalizing the serum D-dimer, FDP, CA125, and CA19–9 levels for the last 2 years.

## Discussion and conclusions

Here we report a 44-year-old woman who developed multiple CIs after her menstrual phase, when the serum D-dimer, CA125, and CA19–9 levels were markedly elevated. Neither warfarin nor novel oral anticoagulants (NOAC) prevented CI recurrence. Although GnRH agonist therapy improved the levels of the three markers and prevented CI recurrence, the CIs recurred after the therapy was discontinued. Hysterectomy finally normalized the serum levels of the markers, and prevented CI recurrence. To our knowledge, this is the first report of an attempt to use NOAC to prevent recurrence of CI associated with adenomyosis. The clinical course of this case suggests that anticoagulation therapy is not sufficient to prevent blood hypercoagulation associated with adenomyosis, and thus resection may be the most effective therapy to normalize hypercoagulation and prevent the recurrence of thrombotic events.

Although reports of CI associated with adenomyosis are rare, some similar cases were reported. Yamashiro et al. [3] reported four cases of CI associated with adenomyosis in middle-aged women. Two of the women developed CIs in their menstrual phase. All of them presented with severe anemia (hemoglobin levels 6.9–8.6 g/dl). The D-dimer levels were elevated in two cases (1.1 μg/ml and 6.0 μg/ml), and CA125 was elevated in three cases (159 U/ml, 42.6 U/ml, and 1750 U/ml). All four cases were treated with antiplatelet medicine or an anticoagulant with a GnRH agonist. One case experienced recurrent multiple CIs after discontinuation of the therapy, when the levels of D-dimer and CA125 again increased (4.1 μg/ml and 907 U/ml, respectively). Subsequently, she was treated with anticoagulant therapy and a GnRH agonist, and the D-dimer and CA125 levels normalized. These authors suggested that hypercoagulability in association with an elevated CA125 level, menstruation-related coagulopathy, or increased tissue factor (TF) expression level is a potential risk factor for developing CI. Hijikata et al. [5] reported 59-year-old woman with a 10-year history of hormone replacement therapy for menopausal symptoms who developed multiple CIs. Her laboratory tests revealed elevated CA125 (334.8 U/ml) and D-dimer (7.0 μg/ml) levels. Anticoagulant therapy with unfractionated heparin was started and the hormone therapy was discontinued. Although antithrombotic therapy was discontinued on day 7 because of withdrawal bleeding, the D-dimer and CA125 levels normalized. The authors concluded that both elevated CA125 levels and hormone replacement therapy are risk factors for hypercoagulability [5].

The mechanism of hypercoagulation initiated by adenomyosis remains uncertain. Several clinical studies reported an association of elevated serum CA125 and CA19–9 levels, and hypercoagulability [3–9]. CA125 is a typical mucin molecule [10], widely utilized for the diagnosis of epithelial ovarian cancer [11]. CA19–9 is a mucin-like high-molecular-weight glycoprotein utilized for the diagnosis of malignancies of the stomach, colon, and pancreas [12], and was recently reported to be associated with thrombosis in pancreatic adenocarcinoma [9]. These cancer-related-mucin molecules are suggested to play an important role in the hypercoagulative state in Trousseau’s syndrome [13]. In our case, the levels of CA125 and CA19–9 were considerably high. We suppose that the high levels of these markers induced the recurrence of multiple cerebral infarctions in our case. A recent experimental study revealed that carcinoma mucin promotes thrombosis through adhesion-dependent, bidirectional signaling in neutrophils and platelets [14]. This mechanism may explain why the hypercoagulation in our case was not prevented by warfarin or NOAC.

In Trousseau’s syndrome, induction of TF and its activity is postulated to promote hypercoagulability in patients with cancer [15, 16]. Expression of TF is also significantly higher in adenomyotic lesions [17], and it renders microparticles procoagulant. Increased release of TF-exposing microparticles is suggested to contribute to the development of thrombotic complications [18]. This pathology might play an important role in multiple thromboses induced by adenomyosis.

Anemia is also a suspected cardiovascular factor [19, 20]. Anemia is considered a hyperkinetic state, and it disturbs endothelial adhesion molecule genes, which may lead to thrombus formation. In addition, anemia causes blood flow augmentation and turbulence, which may result in the migration of a thrombus, thus producing artery-to-artery embolism.

In conclusion, our findings suggest that middle-aged women with adenomyosis are at high risk for CI when the serum D-dimer, CA125, and CA19–9 levels are elevated. In those patients, anticoagulant therapy including NOAC therapy could not prevent CI. Adenomyosis resection may be the most effective therapy for preventing CI in these cases.

## Abbreviations

CA125: carbohydrate antigen 125
CA19–9: carbohydrate antigen 19–9
CI: cerebral infarction
FDG: Fluorine-18–2-fluoro-2-deoxy-d-glucose
GnRH: gonadotropin-releasing hormone
MRI: magnetic resonance imaging
NOAC: novel oral anticoagulants
TF: tissue factor
